# A Comparative Evaluation of the Efficacy of Different Caries Excavation Techniques in reducing the Cariogenic Flora: An *in vivo* Study

**DOI:** 10.5005/jp-journals-10005-1366

**Published:** 2016-09-27

**Authors:** Afrah Fatima Hassan, Gunjan Yadav, Abhay Mani Tripathi, Mridul Mehrotra, Sonali Saha, Nishita Garg

**Affiliations:** 1 Student, Department of Pedodontics and Preventive Dentistry Sardar Patel Post Graduate Institute of Dental and Medical Sciences, Lucknow, Uttar Pradesh, India; 2 Reader, Department of Pedodontics and Preventive Dentistry Sardar Patel Post Graduate Institute of Dental and Medical Sciences, Lucknow, Uttar Pradesh, India; 3 Professor, Department of Pedodontics and Preventive Dentistry Sardar Patel Post Graduate Institute of Dental and Medical Sciences, Lucknow, Uttar Pradesh, India; 4 Professor, Department of Microbiology, Sardar Patel Post Graduate Institute of Dental and Medical Sciences, Lucknow, Uttar Pradesh, India; 5 Reader, Department of Pedodontics and Preventive Dentistry Sardar Patel Post Graduate Institute of Dental and Medical Sciences, Lucknow, Uttar Pradesh, India; 6 Senior Lecturer, Department of Pedodontics and Preventive Dentistry Sardar Patel Post Graduate Institute of Dental and Medical Sciences, Lucknow, Uttar Pradesh, India

**Keywords:** Carbide bur, Caries-affected dentin, Caries excavation, Caries-infected dentin, Dental caries, Polymer bur, Ultrasonic tip.

## Abstract

**Background:**

Caries excavation is a noninvasive technique of caries removal with maximum preservation of healthy tooth structure.

**Aim:**

To compare the efficacy of three different caries excavation techniques in reducing the count of cariogenic flora.

**Materials and methods:**

Sixty healthy primary molars were selected from 26 healthy children with occlusal carious lesions without pulpal involvement and divided into three groups in which caries excavation was done with the help of (1) carbide bur; (2) polymer bur using slow-speed handpiece; and (3) ultrasonic tip with ultrasonic machine. Samples were collected before and after caries excavation for microbiological analysis with the help of sterile sharp spoon excavator. Samples were inoculated on blood agar plate and incubated at 37°C for 48 hours. After bacterial cultivation, the bacterial count of *Streptococcus mutans* was obtained.

**Statistical analysis:**

All statistical analysis was performed using SPSS 13 statistical software version. Kruskal-Wallis analysis of variance, Wilcoxon matched pairs test, and *Z* test were performed to reveal the statistical significance.

**Results:**

The decrease in bacterial count of S. *mutans* before and after caries excavation was significant (p < 0.001) in all the three groups.

**Conclusion:**

Carbide bur showed most efficient reduction in cariogenic flora, while ultrasonic tip showed almost comparable results, while polymer bur showed least reduction in cariogenic flora after caries excavation.

**How to cite this article:**

Hassan AF, Yadav G, Tripathi AM, Mehrotra M, Saha S, Garg N. A Comparative Evaluation of the Efficacy of Different Caries Excavation Techniques in reducing the Cariogenic Flora: An *in vivo* Study. Int J Clin Pediatr Dent 2016;9(3):214-217.

## INTRODUCTION

The most primitive approach for removal of caries was by hand instruments, which was painful and ineffective and led to evolution of rotary instruments like carbide burs.^1^ Carbide burs are indiscriminate in removal of carious tissue, with possible extension into underlying sound dentin.^2^ Henceforth, restorative dentistry has moved away from a “drill and fill” philosophy to a minimally invasive approach.^3^ Very few literature is available on newer techniques like the ultrasonic devices and polymer burs. Hence, the aim of the present study was to compare the efficacy of three different caries excavation techniques in reducing the cariogenic flora.

## MATERIALS AND METHODS

The present study was conducted in the Department of Pedodontics and Preventive Dentistry, Sardar Patel Post Graduate Institute of Dental and Medical Sciences, in collaboration with the Department of Microbiology, Sardar Patel Post Graduate Institute of Dental and Medical Sciences, Lucknow, Uttar Pradesh, India.

A total of 60 healthy primary molars from 26 healthy and cooperative children aged between 6 and 10 years were selected from the outpatient department.

The study design, objectives, potential benefits, and methodology were explained to both children and their parents. Consent and ethical committee clearance were obtained from the Institutional Ethical Committee board prior to the study.

Previously, a pilot study was carried out in the same departments to overview the proper study design and to take care of the possible constraints during the main study.

### Inclusion Criteria

- Clinical criteria:
  – Occlusal carious lesions in vital primary molars.
  – Softened carious lesions involving dentin without extensive coronal destruction.
- Radiographic criteria:
  – Absence of periapical or inter-radicular pathology.
  – Absence of internal or external resorption of roots.
  – Absence of physiological root resorption.

### Exclusion Criteria

- Clinical criteria:
  – Presence of extensive carious lesions (unrestorable teeth).
  – Teeth with history of pain (pulp exposure).
  – Presence of sinus tract, abscess, fistula, or mobility.
- Radiographic criteria:
  – Presence of periapical or inter-radicular radiolu-cency.
  – Presence of internal or external root resorption.
  – Presence of physiological root resorption.

**Fig. 1:**
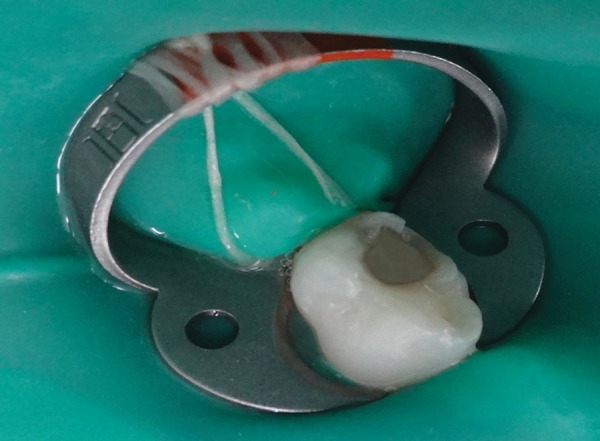
Caries removal by carbide bur

**Fig. 2:**
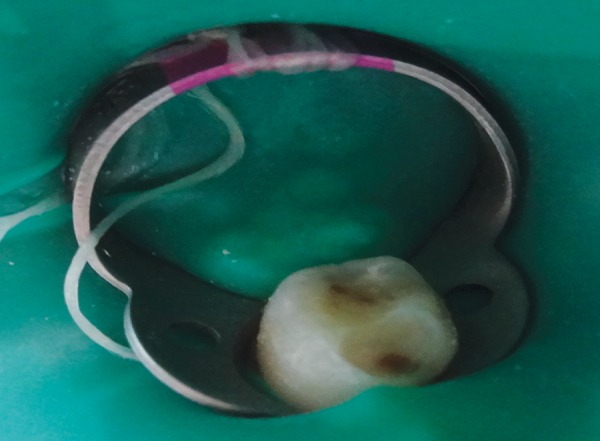
Caries removal by polymer bur

## PROCEDURE

Before beginning the procedure, all the instruments were autoclaved and nonautoclavable instruments were chemically disinfected. Caries excavation was done in 60 occlusal carious primary teeth which were equally divided into three groups of 20 each (n = 20; Group A: carbide bur, Group B: polymer bur, Group C: ultrasonic tip).

**Fig. 3:**
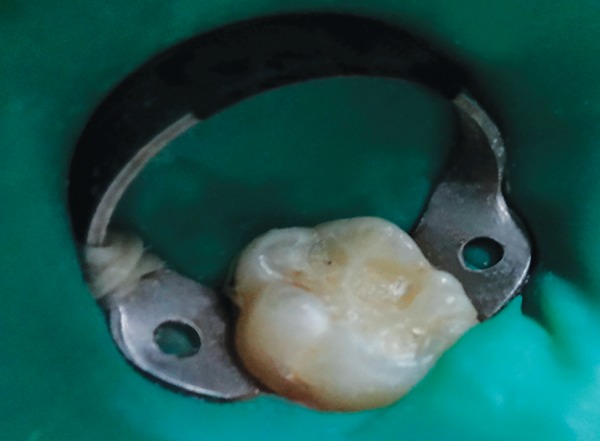
Caries removal by ultrasonic tip

The removal of infected softened carious dentin was done under rubber dam isolation to prevent any contamination of the carious dentin sample (Figs 1 to 3). After caries removal, cavity was assessed by visual and tactile method; and with the help of caries detecting dye for remaining carious lesion. If any part of caries was left, the same procedure was repeated. This was followed by restoring the cavity with glass ionomer cement. Carious dentin sample was taken before and after caries excavation with the help of sterile sharp spoon excavator for microbiological analysis in sterile disposable test tubes containing peptone broth. Samples were taken in sterile conditions and by aseptic technique. The samples were inoculated onto blood agar plates. Next, they were incubated for 48 hours at 37°C for complete bacterial growth.

## COUNTING OF BACTERIAL COLONIES

After 48 hours of bacterial cultivation, the bacterial counts of *Streptococcus mutans* were obtained in colony-forming units (CFUs) and were recorded as CFU/ml × 10^5^. For counting the microbial colonies, magnification glass was used.

## STATISTICAL ANALYSIS

All statistical analysis was performed using SPSS 13 statistical software version. The pre- and posttreatment bacterial counts (CFU/mL × 10^5^) of *S. mutans* were compared by Wilcoxon matched pairs test. The improvements (pre-post) in microbial counts of three groups were compared by Kruskal-Wallis analysis of variance by ranks and the improvement between the groups was by *Z* test. The confidence level of study was proposed to be 95%. Hence, p-value < 0.05 has been considered significant, p-value < 0.01 has been considered highly significant, and p-value < 0.001 has been considered very highly significant.

**Table 1:** **Table 1:** Bacterial count (CFU/ml × 10^5^) of *Streptococcus mutans* before and after caries excavation in different caries excavation techniques

		*Caries before* * excavation*		*Caries after* * excavation*		*Diffirence*		*% reduction*		*Significance*			
*Caries excavation* * techniques*		*Mean±SD*		*Mean±SD*		*Mean ± SD*		*Mean±SD*		*Z-value*		*p-value*	
Carbide bur		8.07 ± 0.80		1.04 ± 0.38		7.03 ± 0.60		87.25 ± 3.77		3.92		< 0.001	
Ultrasonic tip		7.73 ± 0.69		2.03 ± 0.48		5.70 ± 0.53		73.86 ± 5.11		3.92		< 0.001	
Polymer bur		6.68 ± 1.25		2.81 ± 0.75		3.87 ± 0.94		58.05 ± 11.33		3.92		< 0.001	

CFU: Colony-forming units; SD: Standard deviation

**Table 2:** **Table 2:** Between-group comparison of the bacterial count of *Streptococcus mutans*

*Comparisons*		*Z-value*		*p-value*	
Group I *vs* Group II		7.04		< 0.001	
Group I *vs* Group III		3.35		< 0.01	
Group II *vs* Group III		3.69		< 0.01	

## RESULTS

Table 1 shows the bacterial counts before and after the caries excavation in all the three caries excavation techniques. The decrease in the bacterial counts was highest in group I (carbide bur) followed by descending order in group III (ultrasonic tip) and group II (polymer bur).

Table 2 shows the between-group comparison of bacterial count of *S. mutans,* which reveals that the reduction of bacterial counts in group I (carbide bur) was statistically significant as compared with group II (polymer bur) and group III (ultrasonic tip), whereas the reduction was also significant in group III (ultrasonic tip) when compared with group II (polymer bur).

Hence, the order of significance of reduction of bacterial counts of *S. mutans* was as follows:

Group I (carbide bur) > Group III (ultrasonic tip) > Group II (polymer bur).

## DISCUSSION

Caries, because of its uniqueness as a disease, its ubiquitous nature, and its stubborn resistance to resolution, remains as one of man’s most common, oldest, and costliest ailment. The original approach to the treatment of caries was purely surgical.^4^ Conventional caries removal involves the use of a drill on high-speed handpiece to gain access to carious lesions and a low-speed handpiece to remove carious tissue. This method involves quick and efficient caries removal; however, it may result in unnecessary removal of the healthy or even the affected dentin that shows the ability of remin-eralization. This is perceived as unpleasant and painful by many patients and local anesthesia is frequently needed to control pain.^5^

The search for a more gentle, comfortable, and conservative caries excavation has led to the development of methods that aim at providing minimal thermal changes, less vibration and pain, and removal of infected dentin only. In 2003, Boston introduced SmartPrep polymer bur, which is able to distinguish between infected and affected dentin and has the advantage of fewer dentinal tubules being cut, thereby causing less pain sensations.^6^

Recently, the possibility to use ultrasonic instrument for cutting tooth tissue has been introduced.^7^ Moreover, its characteristics for use in pediatric dentistry, such as comfort and the production of a noise, i.e., different from that produced by rotary instruments, may contribute to obtaining a more favorable behavior from children.^8^

Thus, the purpose of this study was to evaluate the reduction in bacterial count before and after caries excavation with different caries excavation techniques, namely carbide bur, polymer bur, and ultrasonic tip.

Cavity preparation prior to restoration requires complete removal of carious dentin. The process is normally deemed complete when the dentin surface appears hard on probing and is stain free.^9^ Determining what is remineralizable and what is not remineralizable dentin is basically a clinical judgment.^10^ In the present study, Canal Blue caries detector dyes were used as clinical guide during caries excavation.

Several investigations have shown that often a low number of residual microorganisms (10^1^-10^4^ CFU) remain behind in clinically sound hard dentin in spite of significant reduction in the bacterial count; however, this low number of bacteria is considered to be clinically acceptable by several authors. Kidd et al studied the relationship of the clinical appearance of carious dentin and the number of bacteria, and they found, as in this study, values below 10^2^ CFU for the total bacterial count for *S. mutans.* The location of residual cariogenic bacteria may also be of significance with regard to secondary caries formation than those situated in other parts of the cavity, though in 1993, Kidd et al found significantly less cariogenic bacteria in hard dentin than in softened dentin. In 1996, she stated that the bacterial count of 1 × 10^2^ CFU/ml after excavation can be considered as the limit for end point.^11^

In the present study, the overall percentage reduction in bacterial count of *S. mutans* was found to be statistically significant in all the three techniques. The percentage reduction in bacterial count was highest in carbide bur, followed by ultrasonic tip and polymer bur. Similar results were obtained by Zakirulla et al^12^, in their study; the percentage reduction in bacterial count was greater in caries removal with carbide bur than with polymer bur and spoon excavator.

The reasons for highest percentage reduction of bacterial count after caries excavation in carbide bur could be due to negative rake angle (design) and less control over the instrument producing nonconservative cavity preparation, which is most likely to be influenced by operator handling.^12^

Caries excavation with polymer bur showed least reduction in bacterial count after caries excavation compared with carbide bur and ultrasonic tip, because it wears off as soon as they contact affected dentin and more than one bur is required to complete the caries excavation. To improve their effectiveness in reducing bacteria, it is suggested to increase their speed and hardness so that they remove carious tissue quicker and with less wear.^12^

Overall percentage reduction of bacterial count with ultrasonic tip was found to be less than carbide bur but greater than polymer bur as ultrasonic tip is considered efficient for cutting hard dental tissue due to its precise cut and its good tolerance by patients. However, there is a need for professional training for the use of this technology since it operates in a different way than do conventional rotary devices.^8^

Though caries excavation with ultrasonic tip showed significant reduction in bacterial count, no data are available about its effectiveness in reducing cultivable bacteria in dentin compared with other caries excavation techniques. Ultrasonic devices are a good alternative, since they do not produce the high-pitched sound that annoys patients; provides better access to cavities and cavity cleaning; demonstrates lower wear of the tips during use; causes less patient discomfort, vibration, sensation of pressure and heat; and are less expensive than lasers.^13^ Further *in vitro* and *in vivo* studies are required to come to a definite conclusion.

## CONCLUSION

- There was decrease in the cariogenic flora in all the three techniques.
- The decrease in cariogenic flora was highest with carbide bur followed by ultrasonic tip and least in polymer bur.
- Ultrasonic tip showed greater reduction in cariogenic flora compared with polymer bur.

Thus it was concluded from the study that carbide bur is most efficient in reduction of cariogenic flora while ultrasonic tip showed almost comparable reduction in cariogenic flora after caries excavation and can be used as a viable alternative for caries excavation.
